# Nematodes join the family of chondroitin sulfate-synthesizing organisms: Identification of an active chondroitin sulfotransferase in *Caenorhabditis elegans*

**DOI:** 10.1038/srep34662

**Published:** 2016-10-05

**Authors:** Tabea Dierker, Chun Shao, Tatjana Haitina, Joseph Zaia, Andrea Hinas, Lena Kjellén

**Affiliations:** 1 Department of Medical Biochemistry and Microbiology, Science for Life Laboratory, Uppsala University, Uppsala, Sweden; 2 Center for Biomedical Mass Spectrometry, Department of Biochemistry, Boston University Medical Campus, Boston, USA; 3 Department of Organismal Biology, Science for Life Laboratory, Uppsala University, Uppsala, Sweden; 4 Department of Cell and Molecular Biology, Uppsala University, Uppsala, Sweden

## Abstract

Proteoglycans are proteins that carry sulfated glycosaminoglycans (GAGs). They help form and maintain morphogen gradients, guiding cell migration and differentiation during animal development. While no sulfated GAGs have been found in marine sponges, chondroitin sulfate (CS) and heparan sulfate (HS) have been identified in Cnidarians, Lophotrocozoans and Ecdysozoans. The general view that nematodes such as *Caenorhabditis elegans*, which belong to Ecdysozoa, produce HS but only chondroitin without sulfation has therefore been puzzling. We have analyzed GAGs in *C. elegans* using reversed-phase ion-pairing HPLC, mass spectrometry and immunohistochemistry. Our analyses included wild type *C. elegans* but also a mutant lacking two HS sulfotransferases (*hst-6 hst-2*), as we suspected that the altered HS structure could boost CS sulfation. We could indeed detect sulfated CS in both wild type and mutant nematodes. While 4-*O*-sulfation of galactosamine dominated, we also detected 6-*O*-sulfated galactosamine residues. Finally, we identified the product of the gene *C41C4.1* as a *C. elegans* CS-sulfotransferase and renamed it *chst-1* (CarboHydrate SulfoTransferase) based on loss of CS-4-*O*-sulfation in a *C41C4.1* mutant and *in vitro* sulfotransferase activity of recombinant C41C4.1 protein. We conclude that *C. elegans* indeed manufactures CS, making this widely used nematode an interesting model for developmental studies involving CS.

Proteoglycans fulfill essential functions in multicellular organisms^1^. The extended glycosaminoglycan (GAG) chains covalently attached to the proteoglycan core proteins are decorated with sulfate groups in cell specific patterns^2^. These are important for the ability of the GAGs to interact with growth factors and cytokines to modulate signaling and influence cell differentiation and migration during development^3^. The most abundant sulfated GAGs are of two types, heparan sulfate (HS)-related (HS and heparin) and chondroitin sulfate (CS)-related (CS and dermatan sulfate)^2^.

While vertebrates and many invertebrates synthesize GAGs, no GAGs have been identified in plants^4^. Marine sponges, representing an early branch in the metazoan tree, also do not produce GAGs but are dependent on acidic glycoproteins for cell-cell contacts. Hydras, belonging to Cnidaria, have the ability to synthesize CS and HS, and both types of GAGs have also been isolated from Lophotrocozoans such as molluscs, flatworms and annelids^4^,^5^. The ability to synthesize both HS and CS is also shared by *Drosophila melanogaster* and other arthropods of the Ecdysozoa clade. Surprisingly, nematodes that also belong to Ecdysozoa produce HS and vast amounts of non-sulfated chondroitin (Chn), but have for long been considered to lack CS^3^,^4^,^6^,^7^,^8^.

Chn-carrying proteoglycans purified from the nematode *Caenorhabditis elegans* have previously been characterized. Interestingly, their core proteins do not resemble the vertebrate counterparts, pointing to a great diversification during evolution^9^. In contrast, all glycosyltransferases required for Chn biosynthesis are conserved in *C. elegans*, but no orthologues of vertebrate CS sulfotransferase genes or of the C5 epimerase required for dermatan sulfate biosynthesis have yet been identified^3^,^10^. Similar to their role in vertebrates, GAGs are essential for the embryonic development of *C. elegans* and RNAi silencing or mutation of *sqv-5*, the Chn synthase, results in severely disturbed early embryogenesis and vulval morphogenesis^11^,^12^. Simultaneous depletion of two of the Chn-proteoglycan core proteins^9^ and loss-of-function mutations in any of seven other genes (*sqv-1, −2, −3, −4, −6, −7, −8*), encoding proteins important for GAG biosynthesis, gave the same phenotype^12^.

It is important to note that the structure of HS synthesized by *C. elegans* is similar to that found in more complex organisms even though the GAG biosynthetic machinery is less complex in the nematode^7^,^8^,^13^; In mammals, HS sulfation is carried out by four *N*-deacetylases/*N*-sulfotransferases, one 2-*O*-sulfotransferase, three 6-*O*-sulfotransferases and seven 3-*O*-sulfotransferases^14^ while *C. elegans* only carries single genes for most of these enzymes^13^.

Since nematodes are a sister group of arthropods among the Ecdysozoans, they would be expected to synthesize CS. The presence of CS in *C. elegans* has also previously been indicated both in a histological and a biochemical study^15^,^16^. However, Chn sulfation has escaped detection in most studies^6^,^7^,^8^ resulting in the general view that nematodes lack the ability to transfer sulfate to Chn^3^,^10^. Since several studies in vertebrates indicate that there may be a functional overlap between HS and CS and that reduced sulfation of HS may enhance CS sulfation^17^,^18^, we decided to look for CS sulfation in a *C. elegans* mutant with defective HS biosynthesis^19^. We wanted to test if the altered HS sulfation also would force the nematode to add sulfate groups to its Chn. By applying a modified GAG purification protocol to enrich for sulfated GAGs we were able to detect sulfated CS disaccharides in the mutants but also in wild type *C. elegans* using established GAG analysis methods such as reversed-phase ion-pairing (RPIP)-HPLC^20^ and mass spectrometry^21^ as well as antibody staining. Most importantly, we could also identify a potential CS sulfotransferase with enzyme activity both *in vivo* and *in vitro*.

## Results

### Optimization of the GAG purification protocol

**Figure 1:**
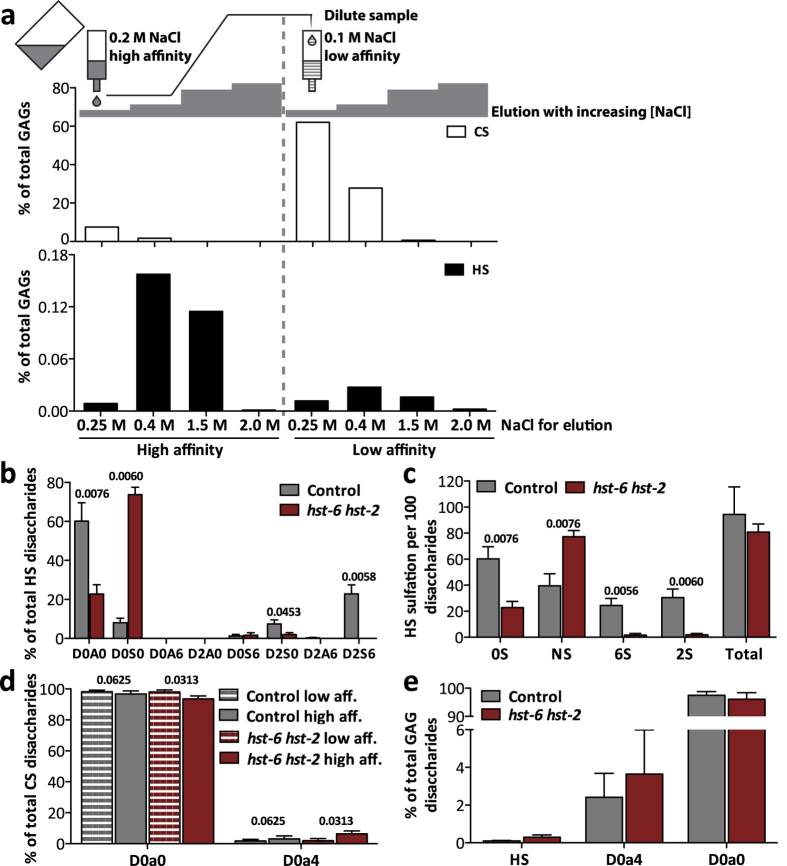
4-*O*-Sulfated CS disaccharides identified by RPIP-HPLC in *C. elegans* GAG preparations. (**a**) Distribution of *C. elegans* GAGs was tested in a pilot experiment: The sample was first adjusted to 0.2 M NaCl and applied to a DEAE column (referred to as high affinity sample). Unbound material was collected, diluted to 0.1 M NaCl and applied to a new DEAE column (low affinity sample). Both columns were subsequently eluted with increasing amounts of NaCl (indicated as grey bars) and total HS and CS recovered were determined by disaccharide analysis on RPIP-HPLC. (**b**) RPIP-HPLC analysis of HS disaccharides from the high affinity fractions of control animals and *hst-6 hst-2* animals (n = 5). The abbreviations used are based on the disaccharide structural code^28^ and refer to the following structures: D0A0 **Δ**HexA-GlcNAc, D0S0 **Δ**HexA-GlcNS, D0A6 **Δ**HexA-GlcNAc6S, D2A0 **Δ**HexA2S-GlcNAc, D0S6 **Δ**HexA-GlcNS6S, D2S0 **Δ**HexA2S-GlcNS, D2A6 **Δ**HexA2S-GlcNAc6S, D2S6 **Δ**HexA2S-GlcNS6S. (**c**) Total sulfation calculated from the results shown in (b) (n = 5). Loss of HS-2-*O*-sulfation (2S) and HS-6-*O*-sulfation (6S) were accompanied by increased HS-*N*-sulfation (NS) in the mutant animals. Total sulfation was not altered. (**d**) RPIP-HPLC analysis of CS disaccharides from the low and high affinity fractions of control and *hst-6 hst-2* animals. The abbreviations used are based on the disaccharide structural code^28^ and refer to the following structures: D0a0 **Δ**HexA-GalNAc, D0a4 **Δ**HexA-GalNAc4S. 4-*O*-sulfated CS disaccharides (D0a4) were significantly increased in high affinity fractions of *hst-6 hst-2* animals compared to low affinity fractions while control animals showed no difference between the fractions (n = 4 for control animals, n = 5 for *hst-6 hst-2* animals). (**e**) The composition of total GAGs (total HS, D0a4 and D0a0) was calculated in control and *hst-6 hst-2* animals (n = 3). Results are presented as mean values, error bars demarcate standard error of mean (SEM), p-values were calculated by one-tailed Mann-Whitney test.

Since non-sulfated Chn is highly abundant in *C. elegans* and may obscure the isolation of sulfated GAGs, a pilot experiment was performed to identify conditions to enrich for HS and CS (Fig. 1a). In this experiment, a wild type *C. elegans* lysate was loaded on a DEAE ion exchange column in 0.2 M NaCl. The GAGs bound to this column were designated “high affinity” fraction whereas the non-binding “low affinity” fraction was diluted to a final concentration of 0.1 M NaCl and applied to a second DEAE column. Both fractions were then eluted in a stepwise manner where the salt concentration was increased to 0.25, 0.4, 1.5 and 2.0 M NaCl. GAGs in all eluates were subjected to enzymatic cleavage into disaccharides, followed by RPIP-HPLC analysis. In accordance with previous studies^7^, HS comprised only a small amount (<0.4%) of the total GAGs in *C. elegans* and more than 80% of the HS was present in the high affinity eluates. As expected, the majority of the Chn/CS was present in the low affinity fraction (Fig. 1a). Interestingly, 10% of Chn/CS was eluted at 0.25 and 0.4 M NaCl from the high affinity column, indicating that some Chn/CS chains may be sulfated. The fact that no Chn/CS was eluted at higher salt concentration indicates however that the degree of CS sulfation is low. Based on the pilot experiment, the conditions for binding of high and low affinity fractions as well as for elution were defined as described in Methods. While further analysis of HS disaccharides was performed on high affinity fractions only, Chn/CS disaccharide analysis was possible to carry out on both high and low affinity samples due to the larger total amounts of Chn/CS.

### Sulfated CS disaccharides identified

Because previous results in zebrafish as well as in mammalian cells have indicated that reduced sulfation of HS may result in increased CS sulfation^17^, we decided to perform GAG analysis on a *C. elegans* mutant lacking the HS-2-*O*- and 6-*O*-sulfotransferases (*hst-6 hst-2* double mutant). Control animals (wild type for *hst-2* and *hst*-6) were analyzed alongside the mutants. HS composition, as determined by RPIP-HPLC, of both mutant and control animals (Fig. 1b,c) was similar to that previously published by others^13^, indicating that our purification method worked satisfactorily. Most interestingly, when Chn/CS composition was analyzed, a peak of 4-*O*-sulfated disaccharides (D0a4) was repeatedly detected in both control and mutant samples (Fig. 1d). This peak was significantly enriched in the high affinity fractions of the *hst-6 hst-2* double mutant compared to the corresponding low affinity fractions (6.4% vs. 2.0%) while no significant enrichment was observed in control animals (3.1% vs. 1.8%). Importantly, adding up the total level of D0a4 in high and low affinity samples from the same starting material revealed that total levels of D0a4 were higher (2.4% and 3.7%) than that of total HS disaccharides (0.1% and 0.3%) in control animals as well as in *hst-6 hst-2* double mutants (Fig. 1e). However, due to variations in the level of D0a4 between experiments, the difference between total HS and D0a4 was not significant for any of the strains.

### Confirmation of CS 4-*O*-sulfation and detection of 6-*O*-sulfated CS with mass spectrometry

**Figure 2:**
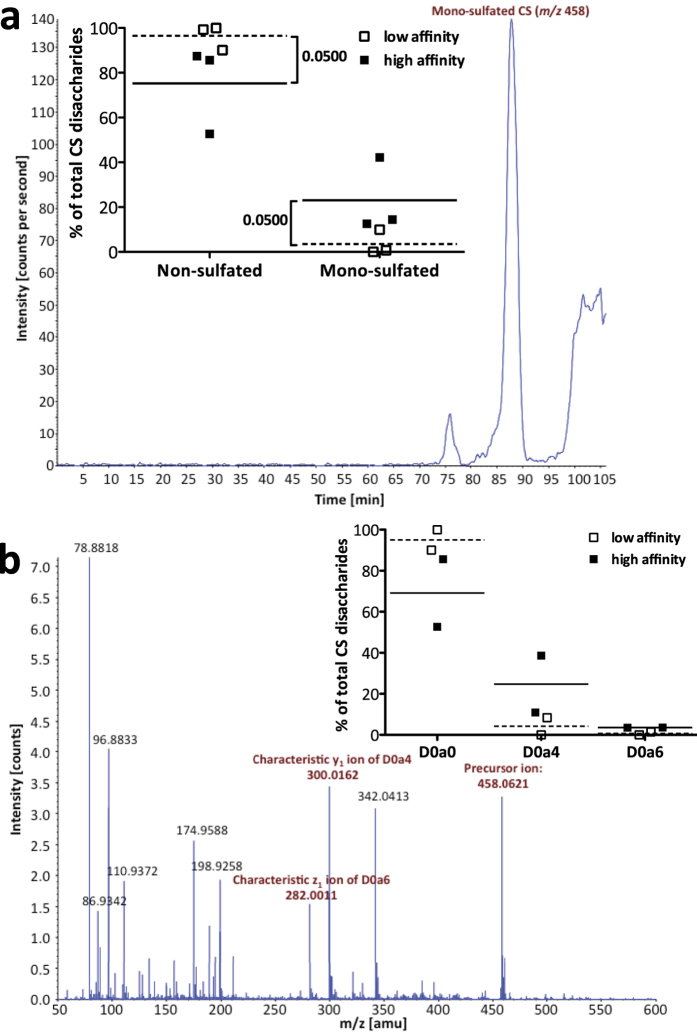
Presence of CS disaccharides confirmed by SEC LC-MS/MS. (**a**) Extracted ion chromatogram of the mono-sulfated disaccharide (*m/z* 458.006 to 458.316) shows elution between 85 and 90 min, similar to commercial standards. The inset shows quantification of non-sulfated as well as mono-sulfated disaccharides (D0a4 or D0a6 (**Δ**HexA-GalNAc6S)) in *hst-6 hst-2* samples (n = 3). Mono-sulfated disaccharides were increased while non-sulfated disaccharides were decreased in high affinity fractions (calculated by one-tailed Mann-Whitney test). (**b**) MS/MS for *m/z* 458 from 85 min to 90 min. Based on the equation of a standard curve calculated for D0a4 and D0a6 mixtures, 76% of the disaccharides in this experiment were estimated to be D0a4 and 24% D0a6. Quantitation from two independent MS/MS experiments using *hst-6 hst-2* samples is shown in the inset. All data points are shown, with the mean indicated.

In order to confirm the presence of the CS disaccharides, we applied mass spectrometry (size exclusion chromatography (SEC) LC-MS/MS) to analyze GAG preparations from mixed stages of *hst-6 hst-2* double mutants. The extracted ion chromatogram for the mono-sulfated disaccharide (*m/z* 458) (Fig. 2a) demonstrated that this ion eluted from the SEC column after 85 min to 90 min, similar to the elution time of mono-sulfated disaccharide standards in the same experiment (not shown). Quantification of three independent GAG preparations revealed the presence of 23.1% ± 9.6% mono-sulfated disaccharides in high affinity fractions and 3.5% ± 3.3% in low affinity fractions (inset in Fig. 2a). The degree of sulfation detected by mass spectrometry was thus strikingly higher than that found by RPIP-HPLC, probably due to an additional purification step by offline SEC prior to chondroitinase (CSase) digestion, which can reduce background noise and removes potential inhibitors of CS digestion.

A mono-sulfated CS disaccharide can either be 4-*O*- or 6-*O*-sulfated. To find out the relative abundance of the two types of disaccharides, tandem mass spectrometry (MS/MS) was applied. The spectrum of the fragmented *m/z* 458 precursor shows both the characteristic y_1_ ion of D0a4 and the characteristic z_1_ ion of D0a6 (Fig. 2b), demonstrating that both 4-*O*- and 6-*O*-sulfation occur in *C. elegans*. Quantification of two independent MS/MS experiments showed 24.8% ± 13.9% D0a4 and 3.6% ± 0.1% D0a6 in the high affinity fractions (inset in Fig. 2b). However, the corresponding low affinity fractions differed strongly, with one showing no sulfation at all and the other containing 8.4% D0a4 and 1.5% D0a6.

### Chondroitin sulfate is mainly present in the cuticle of *C. elegans*

**Figure 3:**
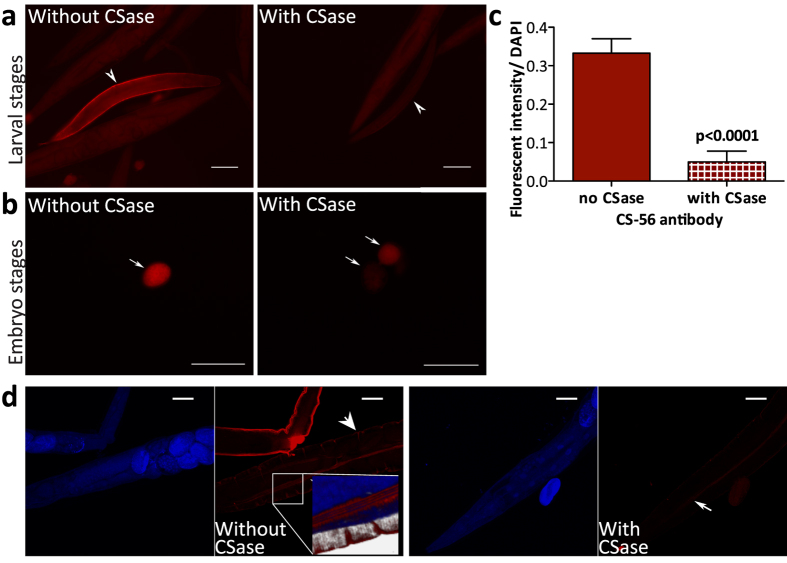
CS distribution during different stages of *C. elegans.* (**a**) Incubation of mixed larval and adult stages of *hst-6 hst-2* animals with the CS-specific antibody CS-56 results in strong staining especially of the cuticle (white arrowhead). CSase ABC treatment prior to antibody detection reduces staining. (**b**) Embryos of *hst-6 hst-2* animals show a strong overall staining (white arrow) with the CS-56 antibody, which can be reduced by CSase ABC treatment. Note that the level of CS-56 signal after CSase ABC treatment varied more for embryos than for later stages. (**c**) Quantification of fluorescent signal intensity from CS-56 staining of mixed adult and larval stages. Values are normalized to DAPI and corrected for non-specific staining. Data from three independent experiments (in total 42 CSase ABC treated worms and 41 untreated worms) are presented as mean values, error bars demarcate SEM, p-value was calculated by one-tailed Mann-Whitney test. (**d**) Confocal images of adult control animals show strong CS-56 staining (red) in the cuticle (white arrowhead), especially in the alae (white arrow head and enlargement of the indicated area), which is greatly reduced by treatment with CSase (white arrow). DAPI staining (blue) indicates the animals’ outlines. Scale bars in all pictures demarcate 100 μm.

In order to confirm our findings and to learn more about the localization of CS in *C. elegans* we stained mixed stages of control and *hst-6 hst-2* animals with the antibody CS-56, recognizing 4-*O*-sulfated and 6-*O*-sulfated CS epitopes but not non-sulfated Chn^22^. Staining was detected in both strains (Supplementary Fig. S1) but was more pronounced in *hst-6 hst-2* animals. In these animals staining was detectable in all stages and was particularly strong in the cuticle of adults (not shown) or at late larval stages (Fig. 3a, white arrowhead) while embryos showed an overall strong staining (Fig. 3b, white arrow). Most importantly, both signals were reduced when the worms had been incubated with CSase ABC, cleaving Chn/CS into disaccharides that are washed away prior to antibody staining (Fig. 3a,b, compare with and without CSase). Quantification of the CS-56 signal intensities normalized to nuclear DAPI staining and corrected for background signal in three independent experiments (42 CSase treated and 41 untreated worms in total) showed a significant signal reduction by CSase ABC treatment (Fig. 3c).

Since the *hst-6 hst-2* animals previously have been shown to display severe developmental defects^23^, wild type *C. elegans* was instead used to localize the CS-56 signal more precisely. Confocal imaging showed that the cuticle of the animals stained strongly with the CS-56 antibody (Fig. 3d), in particular along the alae (white arrowhead and white arrow, respectively, see enlargement for further details). Again, the signal was reduced by treatment with CSase ABC.

### C41C4.1 is an active CS sulfotransferase in *C. elegans*

**Figure 4:**
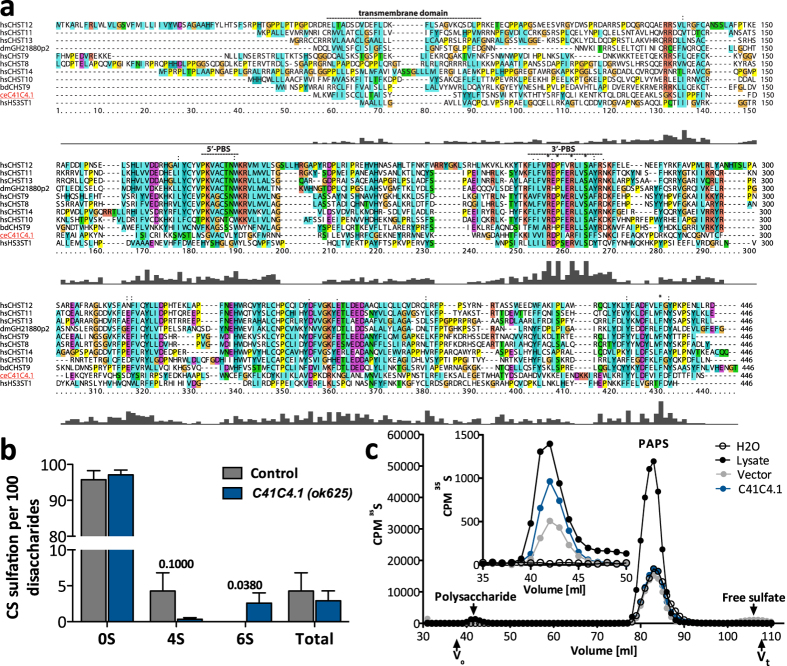
C41C4.1 shows sulfotransferase activity *in vivo* and *in vitro*. (**a**) Multiple sequence alignment constructed with ClustalX 2.1 using the *C. elegans* C41C4.1 protein sequence together with human CHST8 - CHST14 and HS3ST1, GH21880p2 from *Drosophila melanogaster* and predicted CHST9 from *Bactrocera dorsalis.* The predicted transmembrane domain of C41C4.1 as well as its 5′- and 3′-PAPS binding site based on the HNK-1 (CHST10) sequence^30^ are marked with a dashed line. The N-terminal sequences of human CHST8 and CHST9 were truncated during the alignment preparation. (**b**) CS disaccharides from embryonic stages of animals lacking C41C4.1 (blue bars) contain less 4-*O*-sulfate groups but show significantly increased 6-*O*-sulfation while the total sulfation is not markedly affected (n = 3). Results are presented as mean values, error bars demarcate SEM, p-values were calculated by one-tailed Mann-Whitney test. (**c**) *In vitro* CS sulfotransferase activity assay: HEK293 cells stably expressing C41C4.1 or empty pcDNA3.1 vector (control) were lysed and protein was purified as described in Methods. H_2_O served as an additional negative control while unfractionated HEK293 lysate, containing endogenous sulfotransferases, served as positive control. The amount of ^35^S-radioactivity detected in each fraction after separation on a LMW Superdex gel chromatography column is shown, with V_0_ and V_t_ indicated along with the elution position of polysaccharide substrate, PAPS and free sulfate. The insert is a blow-up of the eluted ^35^S-labeled polysaccharide substrate, showing increased ^35^S-incorporation after incubation with the purified C41C4.1 enzyme compared to the empty vector and H_2_O controls.

To date no CS sulfotransferase has been identified in *C. elegans* but based on sequence homology a list of 27 putative CS-6-*O*-sulfotransferases has been published^10^. Abolished or reduced expression of three of these genes resulted in severe phenotypes, suggesting that they play fundamental roles in *C. elegans* development similar to other GAG synthesizing enzymes^11^,^12^. We considered these the best candidates to start searching for active CS sulfotransferases and focused on *C41C4.1* since a mutant strain of this, but not of the other two genes, was available. In contrast to the strains analyzed earlier^10^ the strain *C41C4.1(ok625)* which expresses a C-terminal fragment of the enzyme did not result in a lethal phenotype and could therefore be cultured and used for GAG analysis. Alignment with different human and fruit fly CS sulfotransferases (Fig. 4a) revealed that the amino acid identity of C41C4.1 is highest to human CHST9 (20%) and human CHST10 (17%). In comparison, the fruit fly proteins were more similar to their human counterparts (37% amino acid sequence identity of GH21880p2 from *D. melanogaster* to human CHST13 and 26% identity of the predicted CHST9 from *B. dorsalis* to human CHST9). Binding sites for the sulfate donor 3′-phosphoadenosine 5′-phosphosulfate (PAPS), strictly required for sulfotransferase activity, are conserved in C41C4.1 even though the 5′-PAPS binding site of the C41C4.1 protein displayed a lower degree of conservation, lacking a lysine (K) residue present in the other 4-*O*-sulfotransferases, including the fly proteins. In the 3′-PAPS binding site the RDP motif present in CHST9, CHST10, CHST12 and HS3ST1 is also conserved in C41C4.1 in contrast to the fly proteins, which contain an RHP sequence.

Most interestingly, GAG analysis demonstrated that the amount of D0a4 was strongly reduced in the mutant strain while significantly increased levels of D0a6 were found in these preparations (Fig. 4b). This result suggests that C41C4.1 acts as a CS-4-*O*-sulfotransferase and that 6-*O*-sulfation may be upregulated to compensate for the lowered 4-*O*-sulfation.

In order to directly investigate whether C41C4.1 can act as a sulfotransferase, we expressed the C41C4.1 protein fused to a C-terminal MycHis-tag in HEK293 cells. Expression could be confirmed by qPCR, but was too low to be detected at protein level (data not shown). Importantly, incubation of affinity-purified C41C4.1 protein together with [^35^S]PAPS and the K4 polysaccharide substrate, serving as a Chn substitute as described before^24^, resulted in incorporation of ^35^S-radioactivity into the substrate. Incorporation of ^35^S-radioactivity into the polysaccharide was higher than in the vector control, shown after size-based separation of the samples on a LMW Superdex gel chromatography column (Fig. 4c). Similar results were obtained in two independent experiments after separation on G-25 columns (not shown). We therefore conclude that C41C4.1 is a CS sulfotransferase and rename the *C41C4.1* gene *chst-1* (CarboHydrate SulfoTransferase).

## Discussion

Following a modified purification protocol for GAGs, in which we separated sulfated and non-sulfated GAGs, we could detect 4-*O*- as well as 6-*O*-sulfated CS in *C. elegans*. We speculate that in earlier studies the great abundance of non-sulfated Chn produced by the worm masked the presence of the low-sulfated CS detected in this work^6^,^7^,^8^. In addition, the content of sulfated disaccharides varied strongly between worm cultures when we analyzed mixed stages but was more consistent in embryonic stages of the same strain. Sulfated disaccharides were also difficult to detect in starved cultures that contain few embryos, possibly also contributing to the difficulties in previous studies to detect CS^6^,^7^,^8^.

Using the CS-56 antibody that recognizes 4-*O*- and 6-*O*-sulfated CS, but not Chn^22^, we could show the presence of CS in different developmental stages. As mentioned in the introduction, the presence of sulfated CS was previously suggested based on staining with the cationic dye Cuprolinic Blue^15^. Differences in staining after digestion with CSase ABC and CSase AC, made the authors conclude that dermatan sulfate (consisting of IdoA and GalNAc) was synthesized by the worms as well. Similar to the previous work, we saw a strong signal for CS in the cuticle of the worms (Fig. 3). However, no gene encoding a dermatan sulfate epimerase has so far been identified in the *C. elegans* genome (www.ensembl.org, www.wormbase.org) and CSase B digestion, which acts specifically on DS, did not result in any cleavage of *C. elegans* GAGs^8^, arguing against the presence of dermatan sulfate in *C. elegans*.

When searching for the sulfotransferases responsible for *C. elegans* CS sulfation we identified the uncharacterized protein C41C4.1 as a candidate based on its predicted sulfotransferase activity^10^. Alignment of C41C4.1 with different human and fruit fly sulfotransferases showed the presence of conserved PAPS binding sites in the *C. elegans* protein, supporting the idea that it serves as a sulfotransferase (Fig. 4a). Furthermore, C41C4.1 was less similar to the human HS sulfotransferase HS3ST2 than to the protein family of human carbohydrate sulfotransferases (CHST8–14), which contains enzymes acting both in CS biosynthesis and in sulfation of *N*-linked glycans. Interestingly, C41C4.1 was most similar to human CHST9 (20% amino sequence identity), an enzyme that is not among the main human CS 4-*O*-sulfotransferases (CHST10–14), but has been shown to add sulfate groups to terminal CS disaccharides only^25^, possibly explaining the low degree of sulfation of *C. elegans* CS.

Characterizing the disaccharide composition of the C41C4.1 mutant strain, we found an almost complete loss of 4-*O*-sulfation accompanied by increased 6-*O*-sulfation indicating that this protein acts as a CS-4-*O*-sulfotransferase. The fact that the recombinant enzyme had the capacity to transfer sulfate from PAPS to the chondroitin substrate (K4) supports this notion. However, further studies will be required to characterize the biochemical properties of C41C4.1, now named CHST-1, in more detail.

The increase in 6-*O*-sulfation in the *chst-1* mutant strain indicates the presence of at least one more CS-sulfotransferase in *C. elegans*. However, since the worms have fewer genes encoding HS-synthesizing enzymes than vertebrates, only a few CS sulfotransferases are expected to be expressed by *C. elegans* and their identification is an important next step to elucidate the role of CS in *C. elegans* biology.

Previous studies in *C. elegans* have shown that lack of either *sqv-5*, the Chn/CS synthase, or selected Chn/CS core proteins disrupt early embryonic development^9^,^11^,^26^. Likewise, CSase ABC treatment of mouse embryonic stem cells has been shown to disturb cell division of 2-cell embryos^27^. Since none of these results distinguishes between Chn and CS, the question remains whether sulfation is needed for early embryonic development or if non-sulfated Chn is sufficient. Once the remaining *C. elegans* sulfotransferases have been identified, the nematodes will be a useful system to answer this question.

## Methods

### *C. elegans* maintenance and strains used in this work

Strains were grown on nematode growth media (NGM) agar at 20 °C on *E. coli* OP50. Strains N2, OH4128 (*juIs76; evIs82b*), OH1421 (*hst-6*(*ok273*)), OH1876 (*hst-2*(*ok595*)) and RB813 (*C41C4.1*(*ok625*)) were obtained from the *Caenorhabditis* Genetics Center (CGC). The strains AHS15 (*evls82b*) (referred to as Control) and AHS50 (*evls82b; hst-6*(*ok273*) *hst-2*(*ok595*)) (referred to as *hst-6 hst-2*) were generated from these strains by standard genetic methods. The strain AHS156 (*C41C4.1*(*ok625*)), referred to as C41C4.1(ok625) was generated from RB813 by outcrossing it four times to N2.

Only strains homozygous for the respective mutations were used in this study. All buffers related to *C. elegans* work were prepared according to the recipes on www.wormbook.org.

### Growth of *C. elegans* for GAG analysis

To get large amounts of material for GAG analysis, strains were grown on rich nematode growth medium (RNGM) agar (10–20 plates, 10 cm diameter) seeded with *E. coli* HB101. Worms were grown as mixed stages until the plates were full of worms but not yet starved and were then washed off in M9 buffer. Samples were pooled from all plates, worms were pelleted by centrifugation and then washed in M9 buffer until no traces of bacteria were visible. Worms were then washed with ddH_2_O in order to decrease the remaining amount of salt and freeze-dried. Dry weight was determined.

When GAG analysis was performed solely on embryonic stages of *C. elegans*, worms were grown as mixed stages on RNGM plates as described above and washed off in M9 buffer. Pelleted worms were then incubated in 0.5% NaClO and 1 M KOH until everything but worm eggs was dissolved. Worm eggs were pelleted and washed with ddH_2_O before freeze-drying and determination of dry-weight.

### GAG purification

Purification of GAGs was adapted from previously described methods^20^. Briefly, 30 mg dry material was dissolved in 1 ml protease buffer (50 mM Tris/HCl pH 8, 1 mM CaCl_2_, 1% Triton-X 100). Samples were boiled for 10 min at 100 °C and lysed by consecutively passing them through needles of decreasing diameters. Protease type XIV (Sigma) was added to a final concentration of 0.8 mg/ml and samples were incubated at 55 °C for around 24 h. After heat-inactivation of the protease, samples were adjusted to 2 mM MgCl_2_, before 38 units benzonase (Novagen)/ml were added and samples were incubated over night at 37 °C. Benzonase was then heat-inactivated followed by centrifugation and the supernatants were collected. A pilot experiment was conducted as described under Results to determine the optimal purification conditions. Based on these results NaCl was added to supernatants to a final concentration of 0.25 M NaCl and the samples were loaded on DEAE columns prepared as described^20^. To increase binding, the columns were sealed and incubated for 1 h at 4 °C. The flowthrough fraction was collected, diluted with ddH_2_O to a final concentration of 0.1 M NaCl and applied to a second column, for a 1 h incubation at 4 °C. All columns were subsequently washed with 50 mM Tris/HCl pH 8, 0.1 M NaCl followed by a second wash with 50 mM NaAc pH 4, 0.1 M NaCl (15 min incubation at room temperature each time) and 0.1 M or 0.25 M NaCl (30 min at room temperature). For elution of GAGs, the columns were incubated for 1 h with 1.5 M NaCl at room temperature before the eluate was collected. Depending on their total volume, samples were desalted on NAP-10 columns or PD-10 columns (both GE Healthcare), dried in a SpeedVac and reconstituted in ddH_2_O. CS digestion was performed over night at 37 °C in 0.04 M Tris-acetate, pH 8.0 and approximately 1 mU CSase ABC (Amsbio) per mg dried starting material. Control treatments without addition of enzyme were performed for each sample. After enzyme heat-inactivation the entire sample or a portion of it was subjected to CS analysis via RPIP-HPLC. The other portion was used for isolation of HS, repeating the DEAE chromatography step (without additional time of incubation), desalting via NAP-10 columns and concentration in the SpeedVac. HS digestion was performed over night at 37 °C in 5 mM Hepes pH 7.0, 1 mM CaCl_2_ and 0.4 mU each of heparinase I, heparinase II, and heparinase III (all IBEX Pharmaceuticals), followed by heat-inactivation prior to RPIP-HPLC analysis. Again, control incubations without addition of enzyme were performed for each sample.

### Reversed-phase ion-pairing HPLC

Disaccharides generated by either CSase ABC digestion or by cleavage with a mixture of heparin lyase I, II and III were analyzed by RPIP-HPLC as described previously^20^. Disaccharide standards (Calbiochem) were used to identify and quantify the peaks.

### Size exclusion chromatography (SEC) combined with mass spectrometry (MS/MS) analysis

Samples to be analyzed by mass spectrometry were prepared in the same way as for RPIP-HPLC. Mass spectrometry (SEC LC-MS/MS) was performed on CS disaccharides as previously described^21^. The precursor *m/z* 458 was chosen for tandem MS from 85 min to 90 min elution time from the SEC column, corresponding to the elution time for mono-sulfated disaccharides (D0a4/D0a6^28^).

### Tube fixation and immunofluorescence

One 10 cm NGM plate of freshly starved worms was used for immunofluorescence experiments. Fixation was adapted from the tube fixation protocol described on www.wormbook.org. Briefly, worms were washed off from the plate in M9 buffer, centrifuged for four minutes at 400 × g and kept for five minutes to allow settling of the adult worms at the bottom of the tube. Washing with M9 and centrifugation were repeated until bacteria had been removed and the solution was clear. Finally, worms were washed in ddH_2_O in order to remove any remaining salt. After centrifugation, the pelleted worms were briefly incubated on ice before addition of 1 ml 100% methanol. Samples were frozen in liquid nitrogen until the methanol solidified and then thawed in warm water. Freeze/thaw cycles were repeated four times to ensure efficient cracking of the cuticles. Subsequently, worms were incubated for 1 h at 4 °C, followed by centrifugation for 5 minutes at 5000 × g. After removal of the methanol, samples were washed with PBST (PBS containing 0.05% Triton-X 100) three times. The samples were stored at 4 °C (not more than one week) before continuation of the protocol.

Fixed worms were incubated in PBST with 1% bovine serum albumin (BSA, Sigma) for 1 h at room temperature to block unspecific binding of the antibodies. CS-56 (Sigma), recognizing mono-sulfated disaccharides (D0a4 and D0a6) in a CS chain, was used as primary antibody and Alexa594-anti-mouse (Invitrogen) as secondary antibody. All antibodies were added to the samples in PBST containing 1% BSA and samples were washed after each incubation by vigorous shaking in PBST followed by centrifugation at 400 × g to collect the worms. Finally, worms were resuspended in a small volume of PBST, dropped onto glass slides and mounted with VectaShield (Vector Laboratories Inc.) containing 4′,6-diamidino-2-phenylindole (DAPI) to stain DNA. An antibody control without the primary antibody was included for each experiment.

CSase ABC treatment of fixed worms was performed in 0.04 M Tris-acetate, pH 8.0 in the presence or absence of 100 mU CSase ABC for 3–5 h prior to the first blocking step. After heat-inactivation and centrifugation, the supernatant was removed and immunofluorescence staining was performed as described above.

### Image acquisition and analysis

Worms were analyzed with a Nikon eclipse 90i epifluorescence microscope using Nikon Plan Apo lenses (20x magnification, numerical aperture = 0.75), DS-Qi1Mc camera and NIS-elements (Nikon) software for acquisition. Confocal images were taken on a Carl Zeiss LSM 510 confocal microscope using an LSM T-PMT camera and Zeiss Plan-Apochrome objectives (20x magnification, numerical aperture = 0.8). Confocal z-stacks were visualized with Imaris 7.0 software (Bitplane Scientific Software, Badenstrasse, Zurich).

Quantification of DAPI and CS-56 fluorescent signals was performed using CellProfiler version 2.1.0 (http://www.cellprofiler.org). In total, 42 worms treated with CSase ABC and 41 untreated worms of all stages were quantified in three independent experiments, using 21 (CSase treated) and 25 (untreated) worms without primary antibody as control.

### Recombinant expression and purification of a putative *C. elegans* sulfotransferase

The coding sequence for *C41C4.1* (http://www.wormbase.org/species/c_elegans/cds/C41C4.1#05–10) was amplified from cDNA of mixed worm stages. To this end, total RNA was isolated using TRIzol reagent (Thermo Fisher Scientific) and cDNA was generated applying the iScript kit (Biorad). PCR products generated with the primers C41C4.1 BamHI fw: 5′-aaggatccATGTTGAAATGGTTTATAATTTCTTGTT-3′ and C41C4.1 SacII rv: 5′-aaccgcggGCTATTAATAAAAGTTGTATCAAAATCAAATATTAC-3′ were subcloned into pJET1.2 and their identity was confirmed by sequencing (Eurofins MWG) before they were cloned into pcDNA3.1 myc-HisA (Invitrogen) using the BamHI (*GGATCC*) and SacII (*CCGCGG*) restriction site.

HEK293 cell were transfected with *C41C4.1* in pcDNA3.1 myc-HisA or empty vector using Lipofectamin LTX (Thermo Fisher Scientific) and stable clones were generated by selection with 400 μg/ml neomycin. Successful transfection and selection were confirmed on mRNA expression level by qPCR analysis using the following primers: *C41C4.1 fw*: 5′-AGTCATTCGAGACCCAATTGCT-3′, *C41C4.1 rv*: 5′-AGCATTGCTTACGATCAGGG-3′, and as house-keeping gene *hRPL19 fw*: 5′-CGAATGCCAGAGAAGGTCAC-3′, *hRPL19 rv*: 5′-CCATGAGAATCCGCTTGTTT-3′.

HEK293 cells stably transfected with the C41C4.1 construct or the empty vector were expanded in DMEM Glutamax containing 10% fetal calf serum, 1% PenStrep and 200 μg/ml neomycin (all from Invitrogen). One to two confluent T75 flasks were solubilized in 50 mM Tris-HCl, pH 7.2, 0.8% Triton-X 100, 0.3 M NaCl, 1x Protease inhibitor cocktail cOmplete, EDTA-free (Roche Diagnostics). Recombinant protein was purified using HisPur^TM^ cobalt resin (Pierce) according to the manufacturer’s instructions. The procedure was performed in parallel with vector-transfected cells, serving as negative control.

### CS sulfotransferase activity assay

CS-sulfotransferase activity was assayed in three independent experiments based on described methods^24^. Briefly, similar amounts of total purified protein (C41C4.1 and empty vector) were incubated over night with 5 μg K4 polysaccharide (kindly provided by Anders Malmström, Lund University) and 1 μCi [^35^S] 3′-phosphoadenosine 5′-phosphosulfate (PAPS) in 0.2 M MES, pH 6.5, 10 mM MnCl_2_. After incubation the polysaccharide was precipitated with ethanol as described^29^ and excess [^35^S]PAPS was removed by centrifugation of the samples through Sephadex G-25 columns (superfine grade, Amersham Biosciences) and quantified by scintillation counting. Alternatively, the sample was separated on a LMW Superdex gel filtration column (Pharmacia), separating the ^35^S-labeled K4 substrate from [^35^S]PAPS, followed by scintillation counting.

HEK293 cell lysate containing endogenous CS-sulfotransferases served as positive control and H_2_O as additional negative control.

### ClustalX alignment

Multiple sequence alignment of the *C. elegans* C41C4.1 protein sequence and different human and invertebrate sulfotransferase sequences was constructed with ClustalX 2.1.

### Statistical analysis and data presentation

All graphs were generated using GraphPad Prism version 5.0b for Mac OS X (GraphPad Software, San Diego California USA, www.graphpad.com). Diagrams show mean values, error bars define standard error of mean (SEM). One-tailed Mann-Whitney tests were performed where possible and p-values of 0.05 or less were considered significant.

## Additional Information

**How to cite this article**: Dierker, T. *et al*. Nematodes join the family of chondroitin sulfate-synthesizing organisms: Identification of an active chondroitin sulfotransferase in *Caenorhabditis elegans.* *Sci. Rep.* **6**, 34662; doi: 10.1038/srep34662 (2016).

## Supplementary Material

Supplementary Information
