# Supplementary material for: Nematodes join the family of chondroitin sulfate-synthesizing organisms: Identification of an active chondroitin sulfotransferase in Caenorhabditis elegans
Source: Sci Rep. 2016 Oct 5;6:34662. doi: 10.1038/srep34662 (PMC5050403; doi:10.1038/srep34662)
Supplement: Supplementary Information [file srep34662-s1.pdf]

Nematodes join the family of chondroitin sulfate-synthesizing organisms: Identification of an active chondroitin sulfotransferase in *Caenorhabditis elegans*

Tabea Dierker[1], Chun Shao[2], Tatjana Haitina[3], Joseph Zaia[2], Andrea Hinas[4], Lena Kjellén[1, *]

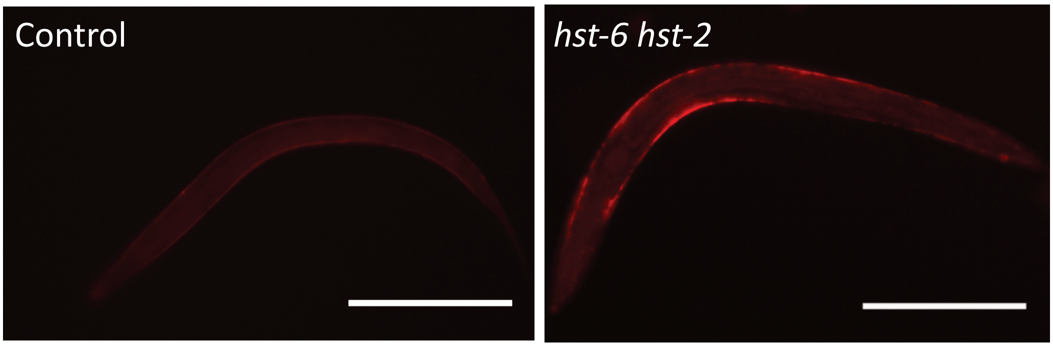

Supplementary Figure S1: CS-56 staining in control and *hst-6 hst-2* animals
Larval stages of control (left panel) and mutant animals (right panel) were stained with CS-56 antibody as described. Staining was visible in both strains but was more pronounced in hst-6 hst-2 animals. Scale bars demarcate 100 µm.
